# Pellicle associated adherence film above incubation broth surface - an inexpensive adjunct to recognizing *Candida krusei *in the laboratory

**DOI:** 10.1186/1756-0500-4-74

**Published:** 2011-03-22

**Authors:** Jacob Fleischmann, Elia M Sripuntanagoon

**Affiliations:** 1 Research Division Greater Los Angeles VA Healthcare System, 16111 Plummer St. North Hills, California, 91343, USA; 2 Clinical Microbiology Laboratory, Greater Los Angeles VA Healthcare System, 11301 Wilshire Blvd. Los Angeles, California, 90073, USA; 3 David Geffen School of Medicine at UCLA, Los Angeles, California, USA

## Abstract

**Background:**

*Candida *species including *Candida krusei *have become common pathogens, especially in immune-compromised patients. Pellicle on the surface of incubating nutrient broth extending with an adherent film above the broth has been described as a feature of this organism. We investigated whether this easily observable adherent film could be useful in the identification of this yeast. We also wanted to see if this process involved any morphological changes from the yeast form on the part of *C. krusei*.

**Findings:**

Common and less frequently isolated species of *Candida *were inoculated into YPD broth and observed for pellicle formation. For *C. krusei *different inoculum sizes and time periods were studied to establish earliest period and the smallest number of organisms needed for this process. A cover-slip assay was established to observe the architecture of the film formed by this organism. Among the clinically common Candida species, only *C. krusei *formed a visible film, requiring 10^6 ^organisms to produce it at 24 hours post inoculation. Film formation also differentiated *C. krusei *from *C. inconspicua *usually reported as a complex by carbohydrate assimilation assays. Rarely isolated *C. famata *and *C. norvegensis *also formed pellicles and film but less robustly. Microscopic observations of the film showed only yeast forms, no hypha or pseudohypha were seen.

**Conclusions:**

Pellicle formation following inoculation of a clinical specimen into liquid culture, is a useful alert to the probable presence of *C. krusei *and likely fluconazole resistance, while awaiting the results of more definitive identification assays. Pellicle and adherence film formation are not a result of polymorphic changes on the part of *C. krusei *as only yeast forms were present.

## Findings

Non-*albicans Candida *species are increasingly isolated from susceptible patients [1] and these include such species as *Candida krusei*, an organism with a high rate of endogenous resistance to fluconazole, making empirical treatment with fluconazole less likely to succeed. Pellicle formation at the liquid-air interface that extends as an adherent film above the surface of the incubating solution, has been described as a feature for this organism [2,3]. We carried out a number of experiments to see if pellicle formation could be used as a characteristic in identifying the likelyhood that *C. krusei *was the infecting pathogen. As this organism exhibits dimorphism such as forming pseudohyphae on corn meal agar [3], we also wanted to see if film formation along the inner surface of the test tube involved a form different from yeasts.

## Methods

A total 80 clinical yeast isolates were obtained from the microbiology laboratories of Greater Los Angeles VA Healthcare System, Ronald Reagan-UCLA Medical Center and Cedars-Sinai Medical Center in Los Angeles. The organisms were identified using standard laboratory methods including API, Vitek and FISH. In addition the following strains were obtained from ATCC: *C. albicans *SC5314 MYA2876, *C. albicans *10231, *C. glabrata *MYA2950, *C. krusei *14243, *C. lusitanea *34449, *C. kefyr *748, *C. famata *2560, *C. norvegensis *201746 and *C. inconspicua *16783. All the yeast were maintained on Sabouraud's Dextrose Agar (SDA) at 4°C and subcultured every four to six weeks.

For assays where the number of inoculated organisms were not counted, a single yeast colony was picked from a fresh SDA plate, re-suspended in 1 ml of YPD (1% yeast extract, 2% peptone, 2% dextrose) and incubated at 30°C for 16 hours in 5 ml tubes (either glass or plastic) without agitation. In experiments to determine the effect of inoculums size on adherent film formation, the inoculums (from fresh liquid culture) was adjusted to the values shown in Table 1 using a hemocytometer. Tubes were observed for adherent film forming above incubating solution.

**Table 1:** Various inoculation doses of *C. krusei *and time to visual film detection.

Time (hrs)	Inoculation Doses (yeast/ml)				
	10^5^	10^6^	10^7^	10^8^	10^9^
**1**	-	-	-	-	-
**2**	-	-	-	-	-
**3**	-	-	-	-	±
**4**	-	-	-	±	±
**5**	-	-	-	±	±
**24**	±	+	+	+	+

- - no band seen in any of 3 experiments

± - band seen in some but not all 3 experiments

+ - band seen in all 3 experiments

For morphological observations, cells were obtained using a sterile loop from the film along the side of the test tube above the culture surface and from culture cell pellet, transferred to a slide and observed by light microscopy at 400× magnification. We also studied the undisturbed architecture of the film as follows. Two alcohol-sterilized 25 mm × 25 mm cover-slips were lowered side by side into a 50 ml conical test tube containing 7 ml of YPD. The conical bottom kept the majority of the cover-slips above the YPD solution. One colony of yeast was inoculated and grown without agitation at 30°C overnight, resulting in a film on the outer side of both cover-slips. They were carefully removed, separated, gently placed on a slide and observed with light microscopy.

## Results

**Figure 1:**
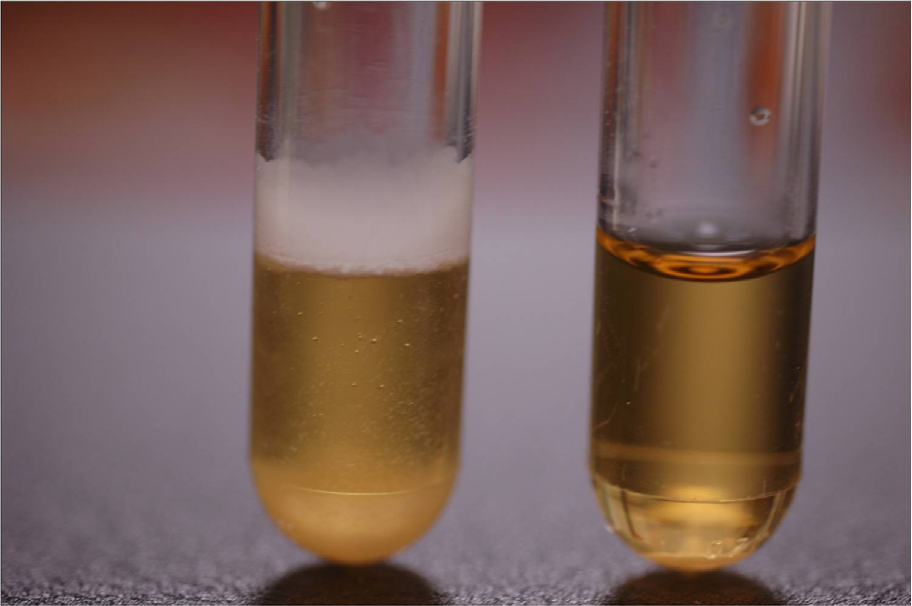
**One colony of yeast incubated in 5 ml of YPD overnight, *C. krusei *in left tube and *C. albicans *in right tube**. A wide film above solution is present in left tube with turbidity of solution visible above settled button. Right tube shows a button at the bottom with clear broth above it with no bands visible above the solution.

Among the more frequently clinically isolated *Candida *species tested, only *C. krusei *exhibited film formation above the broth (Table 2 Figure 1). *C. famata *and *C. norvegensis*, which are only infrequently identified in clinical specimens, also formed adherent films but much narrower than that of all *C. krusei *isolates. Interestingly, carbohydrate assimilation based identification systems such as API usually cannot distinguish *C. krusei *and *C. inconspicua*, but adherent film formation may be able to separate them as the single *C. inconspicua *tested did not form such a film. Use of glass or plastic tubes did not affect pellicle and adherent film formation and it was noted that cultures of *C. krusei *remained much more turbid than tubes containing yeasts not forming pellicles.

**Table 2:** *Candida *species tested for adherence film formation above YPD surface.

Species tested	Number of isolates tested	Film formation
*C. krusei*	15	+
*C. albicans*	10	-
*C. glabrata*	10	-
*C. tropicalis*	8	-
*C. parapsilosis*	12	-
*C. guilliermondii*	3	-
*C. kefyr*	5	-
*C. lusitaniae*	9	-
*C. famata*	2	+
*C. inconspicua*	1	-
*C. utilis*	1	-
*C. lipolytica*	1	-
*C. lambica*	1	-
*C. dubliniensis*	1	-
*C. norvegensis*	1	+

- no adherence film formation after 3 days

+ adherence film formation after 3 days

Various inoculation sizes of *C. krusei *ATCC 14243 were incubated at 30°C for several time intervals and film formation observed (Table 1). At a high inoculum (10^8^/ml) a film was clearly visible in some cultures as early as 4 hours whereas for an inoculum of 10^6^/ml a film was only consistently observed after 24 hours. However, a low inoculum (10^5^/ml) did not consistently form a pellicle and adherent film after 24 hours. In practical terms for all *C. krusei *tested, a single colony picked and incubated formed an easily observable film within 24 hours.

**Figure 2:**
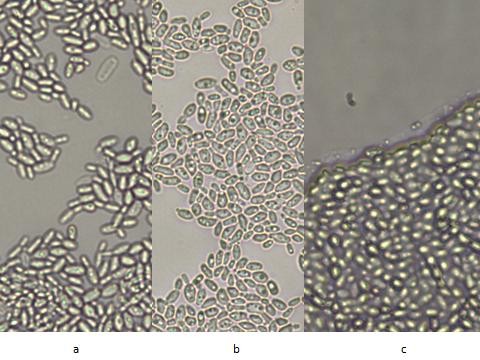
***C. krusei *yeast cells visualized at 400× magnification, a) cells from settled button b) cells scraped from band above solution and suspended in YPD, c) cells growing on cover-slip and visualized without disturbing their pattern**.

We found no differences in morphology between cells obtained from the culture pellet at the bottom of the solution (Figure 2a) and those obtained from the side of the tube above the solution (Figure 2b). In both cases budding yeast cells were seen with some rare elongated cells. In neither did we see any hypha or pseudohypha. Figure 2c represents the undisturbed film that formed on cover-slips above the solution. The figure is in the same orientation as the cover-slip was with the upper part of the figure representing the upper part of the film. One just sees randomly and tightly packed yeast cells and again no hyphal or pseudohyphal elements. At the leading edge a narrow band can be seen possibly representing secreted material by the cells.

Overall, our data shows that pellicle formation is a useful, relatively rapid and inexpensive sentinel for suspecting *C. krusei*, as the other two species forming it, are rare causers of human disease. Furthermore, it adds to the speciation of this organism as it separates it from *C. inconspicua*.

## Abbreviations

YPD: 1% yeast extract, 2% peptone, 2% dextrose; SDA: Sabouraud's Dextrose Agar.

## Competing interests

The authors declare that they have no competing interests.

## Authors' contributions

JF - designed experiments, carried out experiments, wrote manuscript. EMS - collected isolates, carried out experiments. All authors read and approved the final manuscript.
